# Maternal Mortality in Dodoma Regional Referral Hospital, Tanzania

**DOI:** 10.1155/2020/9082179

**Published:** 2020-06-03

**Authors:** Mzee M. Nassoro, Paul Chetto, Enid Chiwanga, Athanase Lilungulu, Deogratius Bintabara, Jacquiline Wambura

**Affiliations:** ^1^Department of Obstetrics and Gynaecology, Dodoma Regional Referral Hospital, Dodoma, Tanzania; ^2^Department of Obstetrics and Gynaecology, University of Dodoma, College of Health Sciences, Dodoma, Tanzania; ^3^Department of Public Health, College of Health Sciences, University of Dodoma, Dodoma, Tanzania

## Abstract

**Background:**

Maternal mortality has remained a challenge in Tanzania. The Tanzania Demographic and Health Survey 2015-16 has shown that the problem has been increasing despite various strategies instituted to curb it. It has been shown that most of the maternal deaths occurring in health facilities, whether direct or indirect, have other contributing factors. The objective of this study was to analyse causes and associated factors for maternal deaths in Dodoma Regional Referral Hospital (DRRH).

**Methods:**

A retrospective review of all files of the women who died in 2018 and were classified as maternal deaths.

**Results:**

A total of 8722 women gave birth in DRRH, out of which 35 died and were confirmed as maternal deaths. The number of live births was 8404 making the maternal mortality ratio of 417 per 100,000 live births. The leading causes of maternal death were eclampsia (9), sepsis (6), ruptured uterus (5), and haemorrhage (5). The third-phase delay was the leading contributing factor to 19 maternal deaths. This includes delays in referral from another facility as well as delays in getting treatment at DRRH and inadequate skills of providers at both the referring facilities and DRRH. The first-phase and second-phase delays contributed to 7 and 6 deaths, respectively. Furthermore, poor antenatal care contributed to 2 deaths.

**Conclusion:**

Maternal mortality is still high in Dodoma Regional Referral Hospital. Eclampsia was the leading cause of maternal deaths in 2018 followed by sepsis and obstetric haemorrhage. Delays associated with health system factors (third-phase delay) contributed much more to maternal mortality than the first-phase delay. Mentorship programmes on management of obstetric complications need to be instituted in order to reduce maternal deaths in Dodoma Regional Referral Hospital.

## 1. Introduction

The World Health Organisation defines maternal death as death of a woman while pregnant or within 42 days of termination of pregnancy, irrespective of the duration and site of the pregnancy, from any cause related to or aggravated by the pregnancy or its management but not from accidental or incidental causes [1]. In this context, one has to be conversant with identifying such deaths; otherwise, there might be undernotification of maternal deaths in health facilities where a woman of reproductive age may die in a medical or surgical ward with an undiagnosed obstetric complication.

Maternal death is an indicator of a poor quality of care. It is for this reason that reduction of these deaths has been a global and national agenda for all countries. During the era of MDGs, goal number 5 was to improve maternal health whereby target number one was to reduce maternal deaths by three-quarters between 1995 and 2015. This target was generally not achieved because there was only 44% decline in the global maternal mortality ratio from 384 to 216 for every 100,000 live births. Ninety-nine percent of these deaths occur in low-income countries and half of these in sub-Saharan Africa [2, 3]. In these countries, the risk of women dying of pregnancy-related complications is 1 in 180, compared to 1 in 4900 for high-income countries [3].

In Tanzania, trends of maternal mortality have remained high. According to TDHS which uses the sisterhood method to estimate maternal mortality, the ratio was 578 per 100,000 live births in 2004-2005 which went down slightly to 454 per 100,000 live births in 2010. However, in TDHS-MIS 2015-16, the ratio had again gone up to 556 per 100,000 live births, a trend which shows that strategies for reduction of maternal mortality have not worked [4, 5].

Dodoma region has been implementing several strategies to reduce maternal deaths as directed in Tanzania Maternal and Perinatal Death Surveillance and Response guidelines, including notifying these deaths within 48 hours and discussing them within 7 days. During such discussions, action plans are devised for identified gaps. In the year 2018, there were 68 maternal deaths recorded in the region, which was 8 more deaths from those of 2017 [6]. Thirty-five of these women died at DRRH.

When complications arise, immediate and appropriate interventions are needed to save the women. By virtue of its status of being a referral centre for the whole region, DRRH sometimes receives women in late stages of obstetric complications due to delays either from home or referring health facilities where care was inadequate. These delays can be categorised in three areas as depicted by Sereen Thaddeus and Deborah Maine. The first-phase delay is at the family level where there is late decision to seek care. This can be due to lack of knowledge of the danger signs, lack of funds to transport the woman to a health facility, or inability of the woman to decide as to where she wants to give birth. The second-phase delay is on the way to reach the health facility. This can be due to lack of transport to a health facility or bad roads. The third-phase delay is when the woman cannot get immediate and adequate care at a health facility either due to shortage or inadequate skills of the staff and shortage of equipment, medicine, or medical supplies [7]. A delayed referral from one health facility to another is also classified as a third-phase delay. These delays are the main contributing factors to maternal deaths. Research has shown that most of the maternal deaths in Tanzania are due to the third-phase delays [8].

As per national guidelines, maternal deaths occurring in DRRH are discussed in order to identify the causes and delays. Unfortunately, these sessions are not attended by all members as outlined in the guidelines on Maternal and Perinatal Death Surveillance and Response. This makes follow-up on the action plans to be difficult. Moreover, no annual analysis of causes of these deaths has ever been done. Despite the small numbers that had occurred in the year 2018, we have decided to make this analysis on the causes and the associated factors and suggest interventions which can help the hospital in reducing these deaths because every one of them counts.

## 2. Methods

### 2.1. Study Design

This is a retrospective case review of all maternal deaths that occurred in Dodoma Regional Referral Hospital in 2018.

### 2.2. Study Setting

The study setting was at Dodoma Regional Referral Hospital in Dodoma city. This is the oldest public hospital in Dodoma region serving as a referral centre for all councils in Dodoma with a population of 2.2 million people. It also serves patients from neighbouring districts of neighbouring regions (Gairo, Morogoro; Kiteto, Manyara; and Manyoni, Singida).

The obstetrics and gynaecology department has five units, namely, antenatal ward, labour and delivery ward, postnatal ward, postcaesarean section ward, and gynaecological ward. There is also a theatre specifically for obstetrics and gynaecology and an intensive care unit which is shared by all departments.

The antenatal and postnatal wards are under the same roof with 32 beds. This ward admits pregnant women at gestation age of 28 weeks onwards with pregnancy-related or any other illness as well as postnatal women with their newborns. The labour and delivery ward admits women in labour either from home, from the antenatal ward, or from other health facilities. There are 5 delivery rooms at the labour ward with 5 delivery beds.

The postcaesarean section ward has 32 beds. It admits patients who had delivered by caesarean section or patients with postpartum complications either from home or from other facilities.

When a patient with obstetric or gynaecological problem is very sick, she is kept in the intensive care unit where doctors from the obstetrics and gynaecology department visit her during the rounds.

The department of obstetrics and gynaecology has five specialists and three registrars. There are also intern doctors who rotate in the department after every three months. The nursing staff consists of registered nurses and nurse midwives. Total deliveries range from 30 to 40 in 24 hours with a caesarean section rate of 22%.

In early November 2018, the department shifted to a new building where all units are now under one roof.

### 2.3. Data Collection

In DRRH, all maternal death files are kept under lock and key after being discussed in Maternal and Perinatal Death Surveillance and Response meetings. The nurse in charge of the department is the custodian of the keys to that locker. All maternal death files from 1st January 2018 to 31st December 2018 were retrieved and analysed. A checklist was used to collect information from the case files. The information collected included age, education status, parity, number of live births, marital status, booking status, referral status, referral diagnosis, length of hospital stay, mode of delivery, interventions in the hospital before death, cause of death, and associated factors.

### 2.4. Data Analysis

Data collected was entered into a computer using Excel programme, and analysis of the entered data was made.

### 2.5. Ethical Considerations

This review was approved by the Dodoma Regional Referral Hospital research committee. Anonymity of the deceased was maintained as required by guidelines when discussing maternal deaths.

## 3. Results

During the year 2018, a total of 8722 women delivered at DRRH and out of them 35 died and were confirmed as maternal deaths. Many of the deceased were 35 years old and above, married, and peasants with no formal education. There were15 grand multipara followed by primigravida (9). Majority of them were admitted as referrals from other health facilities (32) (Table 1).

The main reasons for admission were eclampsia (9), sepsis (6), and ruptured uterus (5). Others were postpartum haemorrhage, anaemia, and peripartum cardiomyopathy, each contributing to 3 admissions. Heart disease in pregnancy, antepartum haemorrhage, abruptio placentae, and intrauterine fetal death contributed to one admission each. Two admissions were due to abortion complications. The leading causes of maternal death were eclampsia (9), sepsis (6), ruptured uterus (5), and haemorrhage (5) (Table 2).

The third-phase delay (delayed referral from other facilities, delay in getting treatment in DRRH, and inadequate skills of providers at a referring facility as well as at DRRH) was the leading contributing factor to 19 maternal deaths. This was followed by delayed decision at the family level contributing to 7 deaths and delays to reach a health facility contributing to 6. Another contributing factor was poor antenatal care (2 deaths) (Table 3).

## 4. Discussion

Maternal mortality is one of the indicators of quality of care in a country and a nation as a whole. It is for this reason that it was in both the Millennium Development Goals (MDGs) and the Sustainable Development Goals (SDGs).

Various methods are used to assess maternal mortality and quality of maternity care in Tanzania. Of these, the Demographic and Health Survey has been used frequently to measure the country's progress in improving maternal and child health. However, these are community-based surveys and information is collected from relatives of the deceased [5]. Moreover, they do not seek to identify the causes of maternal deaths. On the other hand, facility-based analyses go further beyond the numbers and try to establish the cause of maternal death as well as other associated factors including gaps in provision of care and delays.

In the period of this analysis, a total of 8722 women delivered at DRRH and 35 died from causes directly or indirectly related to the pregnancy, delivery, or their management. Moreover, most of these deaths were associated with other factors. Among the deliveries, there were 8404 live births [6]. If extrapolated to the standard reporting of maternal mortality ratio, this is equal to 417 deaths for every 100,000 live births. This ratio is almost similar to that of other referral hospitals such as Shinyanga with the rate 449 per 100,000 live births in 2014 and Kilimanjaro Christian Medical Centre which was 492.1/100,000 as a ten-year trend. Comparably, the national hospital, Muhimbili, has even a higher ratio of 1541/100,000 for the year 2011 [9–11]. These ratios are high and reflect poor quality of obstetric care.

This analysis shows that majority of the deaths occurred in women aged 35 years and above (13 deaths) followed by age groups of 30 to 34 and 20 to 24, both of which contributed to 14 deaths. However, if aggregated, it is obvious that most deaths occurred in women who were 30 years and above (20 deaths). The adolescents and those aged 25 to 29 years had the least contribution to the deaths, each making up 4 deaths. This contradicts the general theory that adolescent pregnancy carries more risks of death because of more unfavourable obstetric risk factors inherent with this age group. These findings are similar to those from a study by Nove et al. and that by Blanc et al. [12, 13].

Grand multiparity has long been established as a risk factor for maternal death. In this analysis, 15 of the deceased were women who were para 5 and above. However, this finding does not correlate with those of Shinyanga Regional Referral Hospital and Muhimbili National Hospital (MNH) where the grand multipara made up 28.3 and 12.1, respectively [9, 10]. This difference could be due to numbers of grand multipara becoming pregnant, especially for big cities like Dar es Salaam.

The major cause of maternal deaths was eclampsia which was responsible for 9 of all maternal deaths. This corresponds to the findings in a study conducted at Bugando hospital in Mwanza where severe preeclampsia and eclampsia caused the majority of deaths [14]. Deaths from eclampsia can be prevented at different levels in the continuum of obstetric care. At the antenatal clinic, blood pressure recordings and urinalysis for proteinuria can identify women with hypertensive disorders and appropriate management can be initiated before worsening of the condition and leading to eclampsia and possible death. However, this is not the case in most of the antenatal clinics in Dodoma. Inadequate antenatal care in detection of hypertensive disorders of pregnancy has also been reported in a research conducted in Dar es Salaam [15].

The prevailing situation in Dodoma is that of unavailability of sphygmomanometers in most of the antenatal clinics where most pregnant women with hypertensive disorders in pregnancy are missed. There is also shortage of these machines in the antenatal and labour wards of some of the lower health facilities leading to delays in identifying women with hypertensive disorders in pregnancy. Furthermore, all facilities caring for pregnant women are supposed to have staff trained in seven signal functions of basic emergency obstetrics and newborn care, one of which is to administer magnesium sulphate for prevention or treatment of eclampsia. However, shortage of skilled staff has had a negative effect on this intervention which is further compounded by delayed referrals of eclamptic patients.

Sepsis caused 6 of maternal deaths at Dodoma Regional Referral Hospital in 2018. This complication occurs if principles of infection prevention and control are not adhered to during provision of obstetric care. In their study on causes and risk factors for maternal mortality in rural Tanzania, Illah et al. found that sepsis was among the top 3 causes of maternal mortality in Rufiji district. In another study conducted at MNH, sepsis ranked fourth in direct causes of maternal deaths [10, 16].

These findings underscore the importance of ensuring that all health care workers attending pregnant and delivering women should strictly adhere to the infection prevention and control practices.

Obstetric haemorrhage and ruptured uterus contributed to 5 deaths each. Despite several interventions in place to prevent and treat obstetric haemorrhage, deaths caused by this condition have become persistent. In the Global Burden of Disease study conducted in 2015, Kassebaum et al. found that obstetric haemorrhage was the leading cause of direct maternal deaths worldwide [17]. In another study conducted in Ifakara, Tanzania, obstetric haemorrhage ranked second as a cause of maternal death [18]. As haemorrhage due to uterine atony is the leading cause of all obstetric haemorrhages, the hospital needs to ensure that active management of the third stage of labour is instituted to every woman during delivery. Likewise, the health care workers should always be ready and work as a team to intervene in an event of obstetric haemorrhage. This can be achieved if every health care worker at the maternity ward is taught simple life-saving manoeuvres such as abdominal aortic compression, bimanual compression of the uterus, and intrauterine balloon tamponade. There should also be a standard operating procedure for following up women who underwent emergency obstetric surgeries. Availability of safe blood at any time cannot be overemphasised.

Uterine rupture causes death due to haemorrhage. Grand multiparity, fetal malposition, malpresentation, and fetopelvic disproportion are risk factors for uterine rupture especially in grand multipara. A previous uterine scar is another significant predisposing cause for uterine rupture. In this analysis, all maternal deaths caused by uterine rupture either had been referred from other facilities or were from home where they had been labouring for more than 24 hours. There is a need to educate multiparous women as well as those with previous uterine scars not to try to deliver at home. The antenatal clinics and facilities without operating theatres should refer patients with risks of uterine rupture much earlier to higher facilities for continuum of care. The use of partograph should also be emphasised in order to detect any deviation in the progress of labour and take appropriate action.

Complications of abortion still have a significant contribution to maternal deaths. In this analysis, 4 women died from such complications. There were no clues as to whether these abortions were induced or not. These findings are similar to those of Shinyanga Regional Referral Hospital and MNH though with different frequencies [9, 10]. They also correspond to the findings of a study on patterns of hospital-based maternal mortality in Tanzania conducted by Bwana et al. [19].

The third-phase delay, which occurs at the health facility level, contributed to most of the deaths which occurred at DRRH. Delayed referral from the other health facilities, delay in getting treatment at DRRH, inadequate skills of providers at a referring facility, and inadequate skills of providers at DRRH are all third-phase delays. Although delayed referral from another facility could be due to unavailability of transport, the health care providers may also lack the ability to realise the criteria needing referral for continuation of care that is not available at that particular lower facility. This could be contributed to shortage of experienced staff because currently there are so many newly employed health care providers working in lower level facilities. Apart from delayed referral, inadequate skills of a health care provider also contributed to deaths. In one of the cases, a poorly repaired lower segment of the uterus continued bleeding and the patient arrived late at DRRH. Deaths due to inadequate skills also occurred in DRRH where in some of the deaths a junior doctor performing caesarean section failed to control bleeding in uterine atony which led to subtotal hysterectomy; unfortunately, the woman died. In their analysis of maternal deaths in Hunan province in China, Lili et al. also found that improper knowledge and skills of county institutions contributed to most of the avoidable maternal deaths [20]. In another study on contributing factors to maternal deaths in health facilities conducted in Nigeria, Burkina Faso, Gambia, Guinea, Senegal, and Sierra Leone, the third delay was found to have a significant contribution [21]. These findings emphasise the importance of adequate skilled staff and availability of equipment in order to prevent avoidable maternal deaths. Availability of means of transport for timely referral is also of paramount importance.

In this analysis, delayed decision at the family level (first-phase delay) contributed to only 7 maternal deaths. This contribution is lower compared to other studies. In a study conducted in Varanasi, India, the first delay was found to have caused 90% of maternal deaths which occurred in that area [22]. Though we could not find the reasons for the first-phase delay in our setting, a study conducted in Bangladesh identified several causes such as delivery conducted by the untrained birth attendant or family members, delays in understanding about maternal complications, delays in decision-making to transfer the mother, and lack of proper knowledge and education, and traditional myth influenced the maternal deaths [23]. These reasons are closely related to lack of women empowerment and also contributed to the delay in reaching a health facility which, in our study, caused 6 deaths. Further contributors to the delay in reaching health facility were lack of funds and transport. Continued advocacy, incentives to traditional birth attendants to bring pregnant women to health facilities, and individual birth plans may reduce maternal deaths caused by the first- and second-phase delays.

## 5. Conclusion

Maternal mortality is still high in Dodoma Regional Referral Hospital. Eclampsia, sepsis, and obstetric haemorrhage were the leading causes. Delays associated with health system factors (third-phase delay) contributed much more to the maternal deaths than the delay in decision-making at a family level (first delay). Delayed referral from other health facilities, delay in getting treatment at DRRH, and inadequate skills of providers at both DRRH and referring facilities have been featured out as the leading associated factors. In order to reduce these delays, timely feedback to all referring facilities needs to be strengthened as well as mentorship programmes to health care workers of these facilities. The hospital should conduct job trainings and regular drills on management of all obstetric emergencies. The senior doctors should also supervise the junior doctors, especially in women with obstetric emergencies requiring surgical intervention. The antenatal, postnatal, and labour wards should have trays with all medicine and medical supplies essential for management of obstetric emergencies such as haemorrhage and eclampsia. All lower health facilities should have clearly structured referral protocols for transportation of women with obstetric emergencies to Dodoma Regional Referral Hospital. A further study on trends of maternal deaths at this hospital is needed to follow up on progress of the interventions instituted.

## Figures and Tables

**Table 1:** General information.

Characteristic	Number
Number of all women who delivered	8722
Number of maternal deaths	35
Number of women who delivered at DRRH	13
Number of women who were referred after childbirth	15
Number of women who died undelivered	4
Mode of delivery at DRRH:	
SVD	2
CS	8
Laparotomy for ruptured uterus	3
Age (years)	
≤15-19	4
20-24	7
25-29	4
30-34	7
35 and above	13
Level of education	
No formal education	18
Primary	14
Secondary	1
College	2
Marital status	
Single	2
Married/cohabiting	33
Occupation	
Housewife	13
Peasant	17
Petty trade	3
Employed (formal)	2
Parity	
1	9
2	6
3	3
4	2
5+	15
Source of admission	
Home	3
Referred from another health facility	32

Of 8722 women who delivered at DRRH, there were 8404 live births and 35 maternal deaths. This makes MMR for 2018 to be 417 per 100,000 live births. Most of the deceased were of age 35 and above and with no formal education. Housewives and petty traders made majority of these women. A great majority of the women who had died were referred from other health facilities.

**Table 2:** Reason for admission and causes of death.

Reason for admission	Number
IUFD	1
Antepartum haemorrhage	1
Abruptio placentae	1
Ruptured uterus	5
PPH	3
Eclampsia	9
Sepsis	6
Anaemia in pregnancy	3
Heart disease in pregnancy	1
Abortion	2
Peripartum cardiomyopathy	3
Cause of death	
Direct causes obstetric	31
Haemorrhage	5
Ruptured uterus	5
Eclampsia	9
Sepsis	6
Abortion complications	4
Peripartum cardiomyopathy	2
Indirect causes	4
Anaemia	2
HIV and AIDS	1
Valvular heart disease	1

There were a variety of causes of admissions, but eclampsia, sepsis, and uterine rupture were the top most. However, if uterine rupture is included in the cause of death due to haemorrhage, then obstetric haemorrhages is the leading reason of admission for those who had died. All haemorrhages (obstetric haemorrhage and ruptured uterus) caused 10 maternal deaths.

**Table 3:** Associated factors contributing to deaths.

Factor	Number
Delayed decision at the family level	7
Delays in reaching the health facility	6
Delayed referral	6
Delay in getting treatment at DRRH	6
Inadequate skills of providers at DRRH	5
Inadequate skills of providers at a referring facility	2
Poor antenatal care	2

Delay in receiving care at the facility level was the leading associated factor. This includes delayed referral from peripheral facilities to DRRH, delayed treatment at DRRH, and inadequate skills of providers at peripheral health facilities as well as at DRRH.

## Data Availability

The data used to support the findings of this study are included within the article.
